# From evidence‐based to sustainable healthcare: Cochrane revisited

**DOI:** 10.1111/jep.13698

**Published:** 2022-05-15

**Authors:** Henrik Berg, Clemet Askheim, Kristin M. Heggen, Tony J. Sandset, Eivind Engebretsen

**Affiliations:** ^1^ Faculty for Psychology, Department of Clinical Psychology University of Bergen Bergen Norway; ^2^ Centre for Sustainable Healthcare Education University of Oslo Oslo Norway; ^3^ Faculty of Health and Sport Sciences University of Agder Kristiansand Norway

**Keywords:** clinical governance, evidence‐based medicine, health policy, medical ethics, philosophy of medicine, science

## Abstract

Evidence‐based healthcare is the prevailing model for healthcare services. In Cochrane's seminal thinking, political context was included with the purpose of promoting healthcare equity. However, the subsequent evidence‐based healthcare models marginalized political context. In this paper, we argue that current models of evidence‐based healthcare fail to respond to emerging healthcare challenges. We claim that reintegration of political context is crucial to make healthcare sustainable. Global communities are anticipating ecological crises with immense repercussions for healthcare. This prospect illustrates that healthcare models failing to integrate political context also risk neglecting some of the most relevant healthcare issues of our time.

## 1 INTRODUCTION

Healthcare is a problem‐solving activity. A crucial question, however, is how we define and demarcate a healthcare problem. Since the early 1990s evidence‐based healthcare has prevailed.^1^, ^2^, ^3^ A major shortcoming of evidence‐based healthcare models is their failure to include political context (i.e., the normative ideals and public decision‐making in a given society). Accordingly, evidence‐based healthcare runs a serious risk of ignoring urgent political issues relevant to healthcare. The sustainable development goals show the need for more encompassing conceptualizations of healthcare. In the sustainable development goals, healthcare is entangled with economic, social, technological, and environmental factors. Hence, the sustainable development goals can relate healthcare to some of the major current political issues. The relevance of including such elements in our understanding of best healthcare has been illustrated by the COVID‐19 pandemic. In this paper, we challenge three underpinning presuppositions in evidence‐based healthcare to make way for sustainable healthcare. These presuppositions are tied to:

1. Temporality: The timelines are included in our understanding of the best practice.
2. Complexity: The systems included in our understanding of the best practice.
3. Values: The values included in our understanding of the best practice.

### 1.1 The marginalization of political context: From cochrane to evidence‐based medicine

Archie Cochrane's *Effectiveness and efficiency* stimulated a revolutionary transition from pathophysiological medicine to evidence‐based medicine. Whereas pathophysiological medicine trusted the expert to have the knowledge and skills to provide the best treatment for the individual patient, Cochrane argued that medical practice should derive more or less directly from scientific evidence. Cochrane provided two concepts to indicate healthcare quality. Effectiveness denotes treatment forms with scientific support using randomized controlled trials. Efficiency refers to the clinical circumstances, the translation of knowledge into practice, and the economic costs tied to different treatment alternatives. In combination, effectiveness and efficiency indicate the rationality of a treatment form and simplifies the comparison of different treatment alternatives.^4^

Cochrane wanted to base medicine on randomized controlled trials because of their lack of bias. He believed a properly conducted randomized controlled trial could indicate causal relationships. However, it is sometimes forgotten that political ideals underpinned Cochrane's vision for healthcare services. In the introduction of ‘Effectiveness and efficiency’  he recalls his enthusiasm for the NHS under the heading: ‘All effective treatment must be free’.

Cochrane aimed at counteracting economic inequalities' impact on the quality of healthcare. In other words, he envisaged an effective public healthcare system available for everyone. Jensen^5^ argued accordingly that Cochrane considered randomized controlled trials as a ‘means to disclose ineffective treatments, and in that way get more money for care to overcome inequalities in the health‐care system’.^5^ Thus, according to Jensen,^5^ Cochrane used scientific evidence as a tool for his political aims. When Cochrane wrote ‘Effectiveness and efficiency’, healthcare services were unevenly distributed and, thus, randomized controlled trials were the vehicles for his political healthcare visions. Importantly, ‘Effectiveness and efficiency’ was originally written as a commissioned evaluation of the NHS. As such, it was a political document serving a political purpose.

The first version of evidence‐based medicine was strictly science‐based without sharing the political commitments of Cochrane.^4^ These models focused exclusively on the medical advantages of replacing pathophysiological models with evidence‐based models. However, they did not regard the medical practice as a vehicle for a more just society. Later revisions were expanded to include clinical expertise and patient values, without being able to include context beyond the clinical setting (e.g., patient values or clinical circumstance).^6^, ^7^ These models risk focusing too narrowly on the clinical encounter, without taking the current political context into consideration. Patient treatment can combine evidence, clinical expertise, and patient values without being sustainable (as defined by the sustainable development goals). Some versions of evidence‐based medicine include concepts like ‘justice’ or ‘fairness’, but they fail to specify the meaning of these concepts. Rather, they focus on scientific rigour introducing epistemological concepts like ‘evidentialism’ (i.e., preferring strong over weak evidence) and ‘reliabilism’ (i.e., preferring good knowledge production routines over bad ones). The implicit presumption in these models seems to be that if the evidence is rigorous, then good practice will follow. Sustainable healthcare, in contrast, starts by asking about what kind of medical services it is possible to provide globally, in the long run, and in harmony with the ecosphere. It would bring about new quality parameters deduced from the sustainable development goals. Thus, in many respects, sustainable healthcare is more in line with the medical ideals of Archie Cochrane, including political context. Whereas Cochrane asked himself how medicine could provide effective healthcare services to everyone, sustainable healthcare asks how healthcare can become equitable, evenly distributed geographically and between generations, and ecologically sustainable.

## 2 SUSTAINABLE HEALTHCARE

The concept of sustainability has a long history and regained relevance when the modern and enlightenment ideals culminated in the industrial revolution^8^, ^9^, ^10^, ^11^ Traditionally, sustainable healthcare services meant services in which resources are sufficient to meet the challenges at hand. In this sense, lack of funding or an ageing population are examples of sustainability challenges in healthcare. However, in this text, and in the current political context, sustainable healthcare is a far more comprehensive concept. Most definitions of sustainability are informed by systems theory and include parameters like ecology, economy, and equity. Models facilitating economic growth will probably affect the ecosphere negatively. Equitable, stable societies are more sustainable than their counterparts.^9^ Reconceptualising healthcare as part of other systems highlights the many existing interlinkages between health and other sections of society.^11^

Unlike similar initiatives, the sustainability development goals have been ratified by all United Nations states. The sustainability development goals include 17 different goals aiming at restructuring life globally. Though often operating at long‐lasting timescales, the problems the sustainable development goals aim at solving are typically urgent. The scientific consensus indicating that we are heading towards disastrous ecological shifts is overwhelming.^12^ When assessing the sustainable development goals it is important to bear in mind that there are no infallible epistemic or ethical grounds for action and that global political consensus containing major reforms is very hard to come by.

It is difficult to envisage that the sustainable development goals could materialize, without including healthcare services. For one, many of the sustainable development goals address healthcare issues directly. Keeping the interlinkages in mind, and provided the vast resources spent on healthcare, healthcare is key to the sustainable development goals. Whereas evidence‐based healthcare aims at providing optimum care for an individual patient, sustainable healthcare aims at providing sustainable care. This entails a reorganization of priorities and contextualizes individual patient treatment to the sustainability goals. Sustainable healthcare respects patient values and autonomy, but patient values and autonomy are expressed within a sustainable framework. In addition, it is very difficult to envisage healthcare being optimal unless it integrates political context and sustainable development goals. Providing efficient treatment according to with individual values at the expense of the ecosphere will neither create better health nor healthcare in the long run.

## 3 FROM EVIDENCE‐BASED HEALTHCARE TO SUSTAINABLE HEALTHCARE

Sustainable healthcare differs from evidence‐based healthcare as developed from the early 1990s and onwards.^2^, ^3^, ^6^, ^7^, ^13^ The former starts from the premise that healthcare practices must be sustainable. In this text, we describe three key parameters to highlight the main principle differences:

### 3.1 Temporality

Sustainable healthcare must include timelines different from those of evidence‐based healthcare. Evidence‐based healthcare conceptualizes best practice according to what has proved to be effective in the past. First, sustainable healthcare practice must include the prospect of future incidents for which we can have no scientific evidence qua randomized controlled trials (although we might have other data strongly supporting sustainable practices). Generally, and to the extent possible, the effects on future generations and the environment should be included in the assessment of the quality of healthcare decisions. The strict methodological schema of evidence‐based medicine is inapt for providing this kind of information. In addition, the tripartite ideal does not include future generations and ecological considerations. Second, sustainable healthcare also entails a reinterpretation of the past. The improvement of the situation of mankind was the backbone of the scientific, technological and industrial revolutions starting in the 16th century.^14^, ^15^ The paradigm of limitless growth, initiated by some of the modern and enlightenment thinkers, must be left for a sustainable paradigm.^11^ Best healthcare does not only aim at finding sustainable ways of reducing disease burdens in the shorter run but for ways compatible with avoiding ecological disasters and the other sustainable development goals.

### 3.2 Complexity

In evidence‐based healthcare, the ideal evidence is scientific propositions indicating the causal effects of single variables. These variables can in turn help us draw relatively clear‐cut inferences about intervention efficacy. In sustainable healthcare practice, the result from randomized controlled trials must be related to a wider context. Whereas the methodological reductionism of randomized controlled trials can be productive, the findings of randomized controlled trials must be utilized in the service of sustainable development goals. Randomized controlled trials can be well‐suited for indicating effectiveness, but not necessarily to demonstrate sustainability. This entails including several systems to assess the sustainability of healthcare practices. It also entails leaving the strict methodological hierarchy for epistemic ideals encompassing more complex relations. Whereas evidence‐based medicine value experimental knowledge to inform medical decision‐making—sustainable healthcare should involve other kinds of evidence. These include, but are not limited to, stakeholder analysis, historical and critical data, and ethical principles. Beyond methodology, the complexity of sustainable healthcare also entails including a vast array of parameters when assessing healthcare services. The 17 different sustainable development goals represent a conglomerate of different aims that should be balanced within and outside healthcare services.

### 3.3 Values

At the one hand, evidence‐based healthcare has clear empiricist leanings often entailing a fact‐value split.^16^, ^17^ At the other, evidence‐based healthcare share the premise of modern and positivist thinkers that science will lead to progress.^15^, ^18^ The most prominent values in evidence‐based medicine are epistemic values (i.e., the goodness of knowledge) and patient values. The typical clinical dilemmas emerge when scientific evidence and patient values diverge.

Sustainable healthcare's point of departure is sustainable values. Science can be a useful tool in the pursuit of these aims, but science will not automatically guide us toward them. Sustainable healthcare is structured according to the values structuring the sustainable development goals. These include, but are not limited to, ecological values, equity, integrity, and dignity. Importantly, parts of the foundation of sustainable healthcare would have to be nonscientific (which is very different from being antiscientific). The precautionary principle is a good example of a nonscientific principle. For some questions, we cannot expect to have (rigorous) scientific knowledge and, in these instances, we should act precautious to minimize risk of severe irreversible harm to the planet and humanity.^19^

## 4 CONCLUSION

The definition of best healthcare practice must include political context. Sustainability issues are urgent, and healthcare practices should play a major role in sustainable societies. Sustainable healthcare practices would not de‐emphasize scientific findings, which could inform deliberations about sustainable healthcare priorities. These deliberations would be a part of much more comprehensive systems thinking and more in line with Cochrane's vision for the improvement of healthcare services. Just like evidence‐based healthcare has changed over the years, we expect the meaning of sustainable healthcare to change. For now, we conclude by stating that sustainability is a precondition for good healthcare services and that healthcare services must be included to realize sustainable societies.

## CONFLICTS OF INTEREST

The authors declare no conflicts of interest.

## Data Availability

There is no empirical data in this paper.
